# Polymorphism-driven coordination geometry engineering for boosting nitrate electroreduction in Cu-pyrazolate chains

**DOI:** 10.1039/d5sc09385f

**Published:** 2026-03-09

**Authors:** Zhanning Liu, Shanna An, Qingzhong Xue, Jian Tian

**Affiliations:** a School of Materials Science and Engineering, Shandong Key Laboratory of Special Epoxy Resin, Shandong University of Science and Technology Qingdao 266590 China znliu@sdust.edu.cn xueqz@upc.edu.cn jiantian@sdust.edu.cn

## Abstract

Tailoring the coordination geometry of metal centers through polymorphism offers a powerful approach to isolating the structural origin of catalytic activity in metal–organic frameworks (MOFs). Herein, two copper-pyrazolate (Pz) polymorphs, α-Cu(Pz)_2_ and β-Cu(Pz)_2_ were synthesized, featuring identical chemical compositions and 1-periodic chain structures but distinct local coordination configurations. Remarkably, the β-Cu(Pz)_2_ exhibits a faradaic efficiency (FE) of 93.33% for the nitrate reduction reaction (NO_3_RR), significantly outperforming α-Cu(Pz)_2_ (53.10%). Comprehensive structural analyses, *in situ* spectroscopy, and density functional theory (DFT) calculations revealed that the coordination geometry governs the electronic structure of the Cu active centers. Specifically, the *cis*-configured β-Cu(Pz)_2_ enables more delocalized Cu 3d orbitals and stronger Cu–O (NO_3_^−^) electronic coupling, thereby promoting nitrate adsorption and activation. This work demonstrates that MOF polymorphism allows precise tuning of electronic structures, offering a fundamental design principle for the development of advanced electrocatalysts toward sustainable nitrogen-cycle chemistry.

## Abstract 2

Coordination geometry-driven polymorphism in copper pyrazolates tailors Cu electronic structures, leading to highly efficient electrocatalytic nitrate reduction to ammonia with a faradaic efficiency of 93.3%.

## Graphical abstract

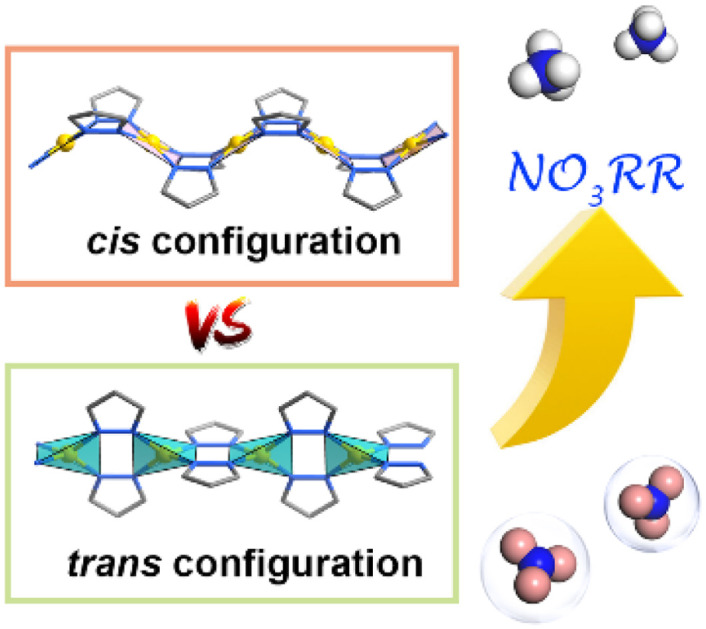

## Introduction

Ammonia (NH_3_) synthesis has been one of the largest chemical industries since the early twentieth century, owing to its crucial role in modern agriculture and its promising potential for energy storage.^1–4^ Among the various strategies for NH_3_ production, electrocatalytic synthesis *via* the nitrate reduction reaction (NO_3_RR) has recently attracted increasing attention as a promising alternative to the energy-intensive Haber–Bosch process.^5–9^ Moreover, by utilizing nitrate as the nitrogen feedstock, this approach integrates energy conversion with environmental remediation, providing a dual benefit for both ammonia synthesis and wastewater treatment.^10,11^ Nevertheless, the reduction of NO_3_^−^ to NH_3_ is a complex process that involves the transfer of eight electrons and nine protons, accompanied by multiple hydrogenation and deoxygenation steps.^12^ This intricate reaction pathway often leads to the formation of various intermediates and by-products.^13,14^ Consequently, the rational design of efficient electrocatalysts and the elucidation of structural-performance relationships remain significant challenges.

Metal–organic frameworks (MOFs), a class of highly crystalline organic–inorganic hybrid materials, have recently emerged as an ideal platform for designing electrocatalysts and conducting mechanistic studies.^15–17^ Their rich chemical diversity, facile functionalization, and modular architecture together provide a robust foundation for elucidating the molecular-level mechanisms underlying electrocatalytic processes. For instance, Cheng *et al.* demonstrated that introducing polar functional groups (–NH_2_ or –NO_2_) into ZIF-7 can enhance CO_2_ capture and activation during electrocatalytic CO_2_ reduction.^18^ Zuo and coworkers reported that a bimetallic Zn_5_-NiS_4_TP MOF shows superior electrocatalytic NO_3_RR performance,^19^ in which the Zn_5_ sites are responsible for reducing NO_3_^−^ to NO_2_^−^, while [NiS_4_] sites within the ligand facilitate the subsequent reduction of NO_2_^−^ to NH_3_. Our recent investigations have demonstrated that tuning the electron-withdrawing strength of halogen atoms (F, Cl, and Br) in the copper anilates can effectively modulate the electronic structure of Cu^2+^, thereby facilitating the deoxygenation of *NO intermediate.^20,21^ However, these strategies typically rely on introducing additional atoms or functional groups, which may complicate or obscure the precise elucidation of the intrinsic active sites.

Polymorphism, referring to the ability of a single compound to exist in two or more distinct crystalline forms,^22–24^ is widely observed in MOFs. Subtle variations in the arrangement or coordination geometry of the building blocks can induce pronounced changes in their physical and chemical properties, offering a powerful platform for unraveling structure-property relationships.^25–27^ For instance, the packing arrangement changes of ligand in cadmium squarates can induce dramatically different thermal expansion behaviors.^25^ In Al-based MOFs, polymorphism arising from *cis-trans* configurations of µ-OH-connected AlO_4_(OH)_2_ chains leads to markedly different water adsorption behaviors.^27^ However, its potential in electrocatalysis remains rarely explored. To this end, Cu^2+^ based MOFs provide an ideal model system, as Cu^2+^ centers not only exhibit a favorable alignment between the d-orbital energy levels and the LUMO π* of NO_3_^−^,^28^ but also display diverse coordination modes that can give rise to rich polymorphic behavior.^29–31^

Motivated by these points and as a proof of concept, we herein systematically investigated the electrocatalytic NO_3_RR performance of two 1-periodic copper-pyrazolate (Pz) polymorphs, α-Cu(Pz)_2_ and β-Cu(Pz)_2_. Remarkably, the β-Cu(Pz)_2_ exhibits a significantly enhanced NO_3_RR activity with the Faraday efficiency (FE) reaching 93.33%, while the α-Cu(Pz)_2_ only delivers a FE of 53.10%. Comprehensive analyses combining long-range and short-range structure investigations, *in situ* spectroscopic measurements, and density functional theory (DFT) calculations reveal that the coordination geometry of Cu centers plays a decisive role in modulating their electronic structures, thereby governing the adsorption and activation of nitrate species and ultimately determining the overall NO_3_RR performance.

## Results and discussion

**Fig. 1:**
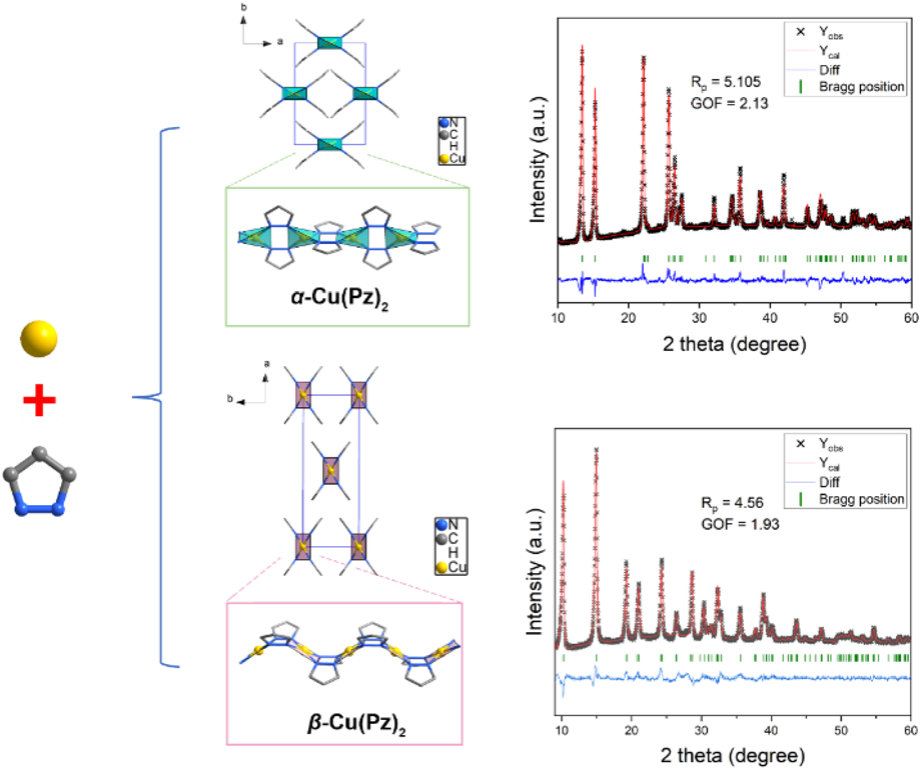
Illustration of crystal structures of α-Cu(Pz)_2_ and β-Cu(Pz)_2_ and the corresponding Rietveld refinement patterns.

The α-Cu(Pz)_2_ and β-Cu(Pz)_2_ phases were synthesized *via* soft-chemistry methods, and the detailed procedures are given in the SI.^32^ Powder X-ray diffraction (PXRD) analyses, combined with Rietveld refinements (Fig. 1), confirmed that both products are phase pure and crystallize in distinct structures. Despite sharing the same chemical formula and both exhibiting infinite one-periodic (1p) chain motifs, their spatial arrangements are dramatically different. As shown in Fig. 1, the 1p chains in α-Cu(Pz)_2_ pack into an orthorhombic phase with the lattice parameters *a* = 7.922 Å, *b* = 11.504 Å, and *c* = 7.791 Å, whereas β-Cu(Pz)_2_ adopts a different unit cell with *a* = 16.970 Å, *b* = 6.236 Å, and *c* = 7.294 Å. The distinct packing modes stem from the local coordination differences within the 1p chain. In both samples, each Cu^2+^ is coordinated by four nitrogen atoms from four Pz ligands. However, in the α-Cu(Pz)_2_ phase, the Pz molecules adopt an alternating *trans* configuration, forming nearly linear 1p chains along the *a*-axis. In contrast, the β-Cu(Pz)_2_ phase displays a uniform *cis* configuration, in which the Pz molecules are oriented in the same direction, giving rise to a zig-zag chain conformation. Such distinct chain geometries induce different steric effects and packing behaviors, ultimately giving rise to the observed polymorphism of the Cu(Pz)_2_ system. The scanning electron microscope (SEM) images (Fig. S1) showed that both samples consist of nanosized particles with diameters in the range of 200–500 nm.

**Fig. 2:**
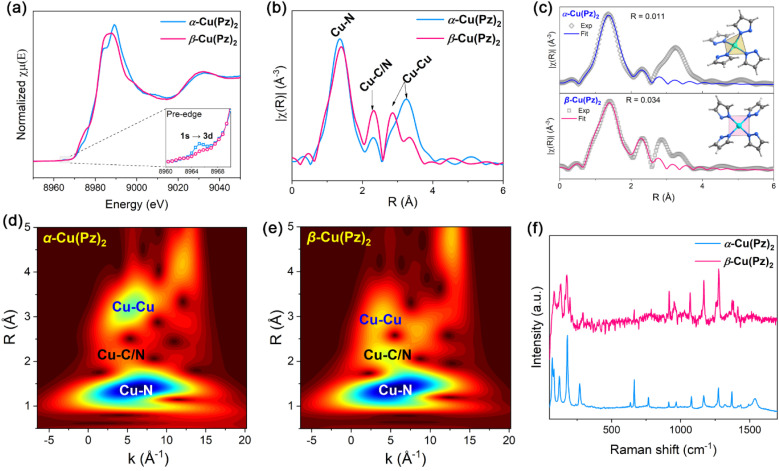
(a) Cu K-edge XANES patterns of α-Cu(Pz)_2_ (blue) and β-Cu(Pz)_2_ (pink), and the inset shows the enlarged pre-edge region. (b and c) EXAFS patterns and the fitted results. (d and e) Wavelet transform contour plots of the two samples. (f) Raman spectra of α-Cu(Pz)_2_ (blue) and β-Cu(Pz)_2_ (pink).

To gain atomic-level insight into the local coordination environments of α-Cu(Pz)_2_ and β-Cu(Pz)_2_ and to minimize potential effects from atomic occupancy disorder, Cu K-edge X-ray adsorption fine structure (XAFS) analyses were carried out. As shown in Fig. 2a, the X-ray absorption near-edge structure (XANES) spectra of the two compounds exhibit nearly identical adsorption edges, indicating that the Cu ions in both samples share similar oxidation states. This conclusion is further validated by the high-resolution X-ray photoelectron spectroscopy (XPS, Fig. S2), which confirms that the Cu ions in both samples are in the +2 oxidation state. Notably, the pre-edge peak at ∼8965 eV is more pronounced in α-Cu(Pz)_2_ than in β-Cu(Pz)_2_. Since this feature arises from the 1s → 3d electronic transition,^33–35^ which is typically intensified in non-centrosymmetric coordination environments, the result indicates that the Cu^2+^ site in α-Cu(Pz)_2_ possesses a less symmetric local coordination environment. This was further supported by the extended X-ray absorption fine structure (EXAFS) analyses. As shown in Fig. 2b, the first peak at ∼1.4 Å corresponds to the Cu–N bond, while the second coordination-shell peak at ∼2.3 Å is attributed to Cu–C/N correlations arising from the next-nearest-neighbor C/N atoms of the Pz ligand. It should be noted that the EXAFS data are not phase-corrected, resulting in an apparent radial shift of approximately 0.4 Å relative to the actual Cu–N distances.^36^ The EXAFS fitting results reveal that Cu^2+^ in both compounds is four-coordinate (Tables S1 and S2). However, the coordination geometries differ distinctly, being a distorted tetrahedron in α-Cu(Pz)_2_ and a square-planar configuration in β-Cu(Pz)_2_ (Fig. 2c). According to the Cambridge Crystallographic Data Centre (CCDC), most of the Cu(ii)-N_4_ based complexes preferentially adopt a square-planar geometry. Thus, the distorted tetrahedral coordination environment in α-Cu(Pz)_2_ may render this phase metastable. Further DFT calculations showed that, the ground-state energy of β-Cu(Pz)_2_ is significantly lower than α-Cu(Pz)_2_ with Δ*E*_0_ = −0.12775 eV (Table S3), confirming the higher intrinsic stability of β-Cu(Pz)_2_. Fig. 2d and e display the wavelet transform (WT) contour plots of the two samples. The lobes observed at ∼5 Å^−1^ and ∼3 Å are assigned to the third coordination sphere, corresponding to Cu–Cu pairs. Obviously, the Cu–Cu distance in α-Cu(Pz)_2_ is longer than that in β-Cu(Pz)_2_, which is consistent with the quasi-linear and zig-zag configurations observed in their long-range structures. Moreover, the Raman spectra of the two compounds show similar characteristic bands (Fig. 2f), validating their identical building blocks.

**Fig. 3:**
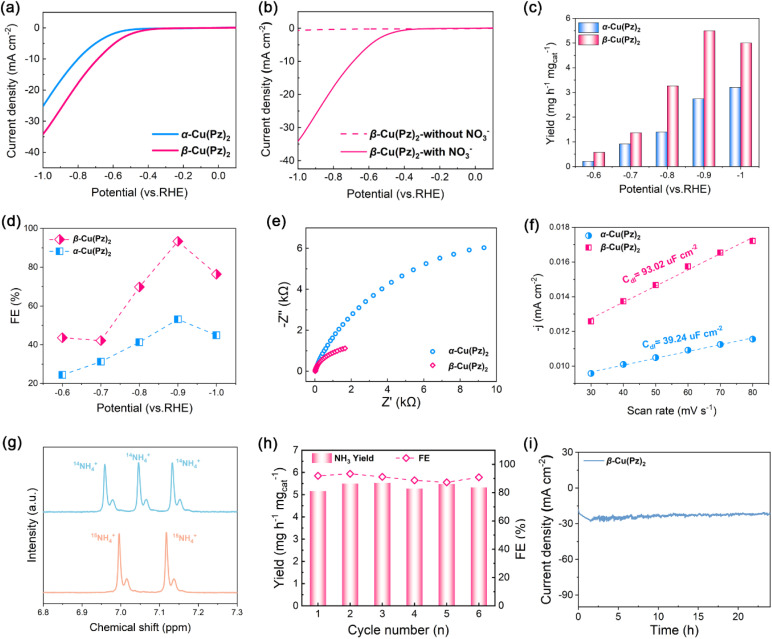
(a) LSV curves for α-Cu(Pz)_2_ (blue) and β-Cu(Pz)_2_ (pink) in the electrolyte. (b) Comparison of LSV curves for β-Cu(Pz)_2_ recorded with (solid line) and without (dashed line) NO_3_^−^. (c and d) NH_3_ yield rates and corresponding FEs of α-Cu(Pz)_2_ (blue) and β-Cu(Pz)_2_ (pink) at different potentials. (e and f) EIS and ECSA measurements of the two samples. (g) ^1^H NMR spectra of the products after NO_3_RR over β-Cu(Pz)_2_ using K^14^NO_3_ (blue) and K^15^NO_3_ (orange) as the feeding nitrogen sources. (i) Cycling stability tests of β-Cu(Pz)_2_ at −0.9 V (*vs.* RHE) for 6 cycles. (h) Time-dependent current density curve at −0.9 V (*vs.* RHE) for over 24 h for β-Cu(Pz)_2_.

Electrocatalytic NO_3_RR performances of the two polymorphs were evaluated in a typical H-type electrolytic cell containing 0.1 M Na_2_SO_4_ and 0.1 M KNO_3_. The linear sweep voltammetry (LSV) curves of α-Cu(Pz)_2_ and β-Cu(Pz)_2_ are shown in Fig. 3a. The current density of β-Cu(Pz)_2_ is obviously higher than that of α-Cu(Pz)_2_ at the same potential, inferring its higher electrocatalytic activity. In addition, the pronounced increase in current density in the presence of NO_3_^−^ confirms its electrocatalytic NO_3_RR performance (Fig. 3b). Based on the LSV results, a series of NO_3_RR measurements were conducted under different potentials ranging from −0.5–−1.0 V *vs.* RHE. The NH_3_ yield rates were quantified using a chronopotentiometry method (Fig. S3–S5). As shown in Fig. 3c and d, both NH_3_ yield rates and FEs display a volcanic trend with increasingly negative potentials. At all applied potentials, β-Cu(Pz)_2_ outperforms α-Cu(Pz)_2_. Remarkably, at −0.9 V *vs.* RHE, β-Cu(Pz)_2_ achieves an NH_3_ yield rate of 5.50 mg h^−1^ mg_cat_^−1^ with an FE of 93.33%, nearly twice that of α-Cu(Pz)_2_ (2.74 mg h^−1^ mg_cat_^−1^ and 53.10%) and outperforming most of the reported copper-based electrocatalysts (Table S3). These results indicate that changes in the local coordination geometry of the microenvironment exert a significant regulatory effect on the catalytic performance. To confirm the origin of ammonia production, isotope labeling experiments were performed. As shown in Fig. 3g, the ^1^H nuclear magnetic resonance (NMR) spectra using ^15^NO_3_^−^ as the feeding nitrogen source display the characteristic doublet of ^15^NH_4_^+^, whereas ^14^NO_3_^−^ produces ^14^NH_4_^+^ with a triplet signal. These results confirm that the NH_4_^+^ detected in the electrolyte does originate from nitrate reduction rather than other impurities. Besides, the electrochemical impedance spectroscopy (EIS) measurements were conducted to assess the conductivity of electrodes. As shown in Fig. 3e, the β-Cu(Pz)_2_ exhibits a significantly lower interfacial electron-transfer resistance. Meanwhile, the electrochemically active surface area (ECSA) measurements (Fig. 3f, S6) indicate that β-Cu(Pz)_2_ has a higher *C*_dl_ value of 93.02 µF cm^−2^, obviously exceeding that of α-Cu(Pz)_2_ (39.24 µF cm^−2^), suggesting a greater number of accessible active sites in β-Cu(Pz)_2_. The cycling performance of β-Cu(Pz)_2_ was evaluated at −0.9 V *vs.* RHE. Over six consecutive cycles, no significant decrease in NH_3_ yield rate or FE can be observed (Fig. 3h), and negligible metal ion leaching was detected. Moreover, the 24 h time-dependent current density profile shows negligible decay (Fig. 3i), further confirming its long-term stability and potential for practical applications.

**Fig. 4:**
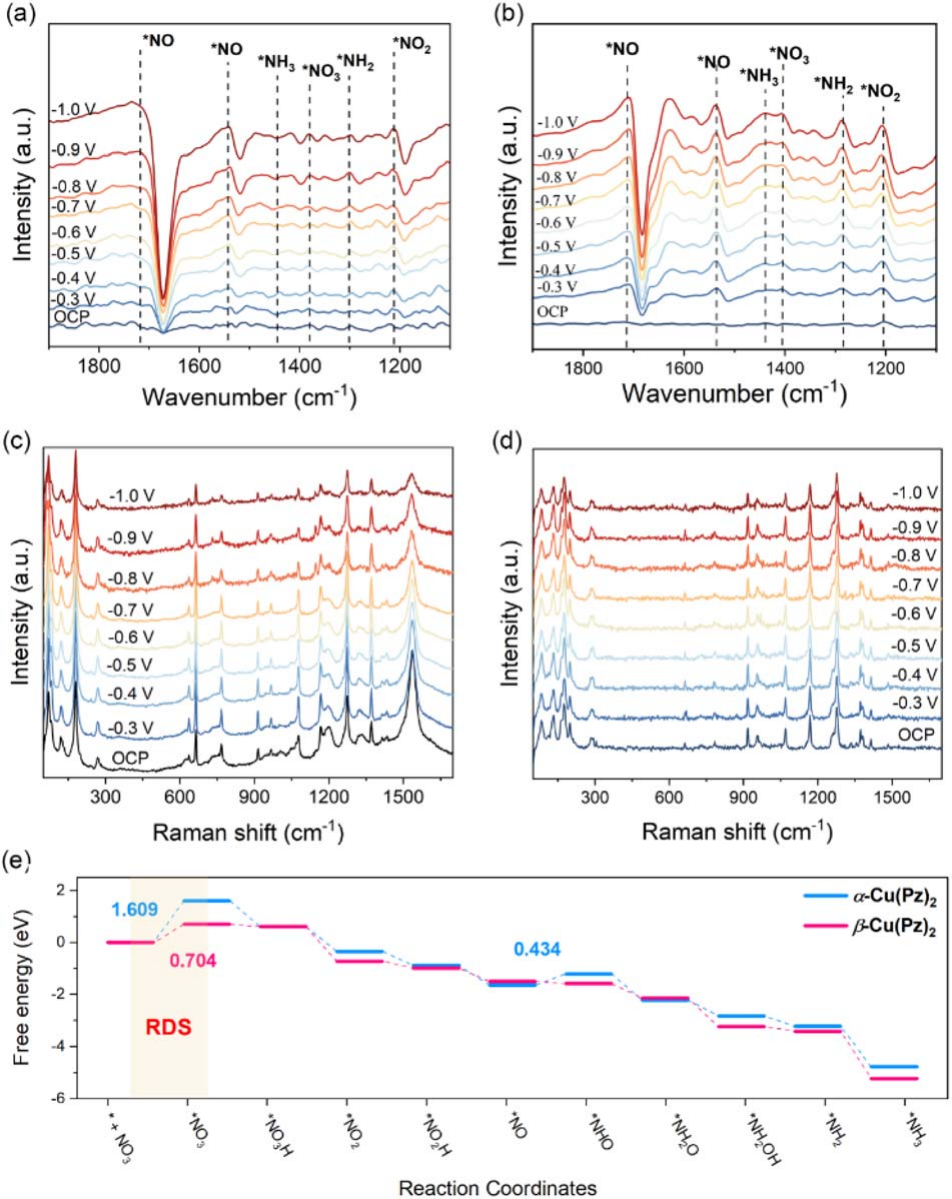
*In situ* FTIR and Raman spectra of α-Cu(Pz)_2_ (a and c) and β-Cu(Pz)_2_ (b and d). (e) Calculated Gibbs free energy diagram along the catalytic reaction pathway on α-Cu(Pz)_2_ (blue) and β-Cu(Pz)_2_ (pink).

*In situ* Fourier transform infrared (FTIR) spectroscopy was employed to probe the possible reaction intermediates formed during the ammonia synthesis process over α-Cu(Pz)_2_ and β-Cu(Pz)_2_ catalysts. As shown in Fig. 4a and b, both catalysts exhibit similar intermediate-related signals as the applied potential shifts from open-circuit potential (OCP) to −1.0 V *vs.* RHE. Among them, the band at 1404 cm^−1^ is attributed to the adsorbed NO_3_^−^ species, while the bands at 1205 cm^−1^, 1537 cm^−1^, and 1285 cm^−1^ correspond to the formed *NO_2_, *NO, and *NH_2_ intermediates,^37,38^ respectively. These characteristic peaks suggest that the electrocatalytic NO_3_RR proceeds through a successive deoxygenation and hydrogenation process. Notably, the intermediate-related IR bands of β-Cu(Pz)_2_ exhibit a markedly higher signal-to-noise ratio than those of α-Cu(Pz)_2_, implying enhanced catalytic performance. This observation is fully consistent with its superior NO_3_RR performance described above. Furthermore, *in situ* Raman spectra were used to detect the possible structural changes in the catalysts (Fig. 4c and d). Under the applied potentials, no additional Raman bands were observed, confirming the structural stability of the catalysts.

**Fig. 5:**
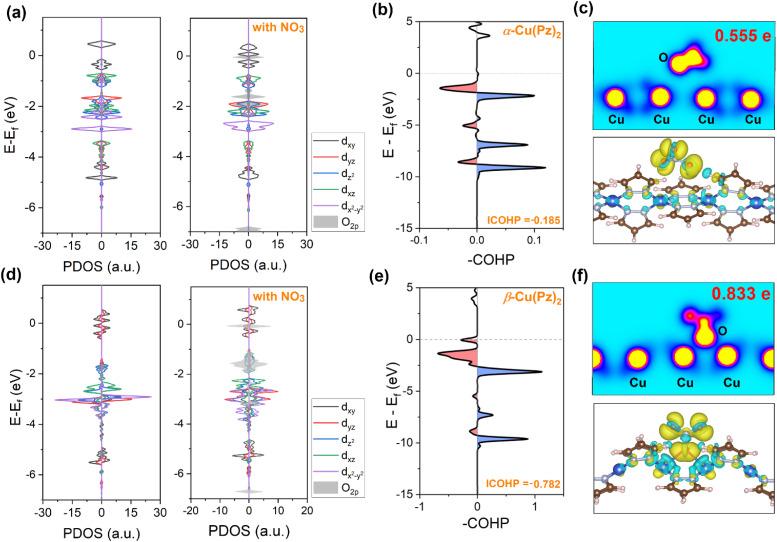
(a and d) Spin-polarized PDOS of α-Cu(Pz)_2_ and β-Cu(Pz)_2_ before (left) and after (right) nitrate adsorption. (b and e) COHP curves and corresponding ICOHP values for the Cu–O bonds. (c and f) Charge density difference cross-sectional slices (top) and isosurface maps of nitrate adsorbed α-Cu(Pz)_2_ and β-Cu(Pz)_2_.

Subsequently, guided by the above *in situ* spectroscopic results, density functional theory (DFT) calculations were carried out to further elucidate the origin of their distinct catalytic activities (Fig. S7 and S8). The electrocatalytic NO_3_RR process primarily proceeds through the following pathway: (1) adsorption and activation of NO_3_^−^ to form *NO_3_ radical; (2) successive deoxygenation to produce *NO and stepwise hydrogenation *via* proton-coupled electron transfer (PCET) to ultimately generate *NH_3_.^39^ As depicted in the free energy profiles (Fig. 4e), NO_3_^−^ adsorption is the rate-determining step (RDS) throughout the overall reaction process for both catalysts. The corresponding energy barrier on β-Cu(Pz)_2_ (Δ*G* = 0.704 eV) is significantly lower than that on α-Cu(Pz)_2_ (Δ*G* = 1.609 eV), indicating a more favourable activation of the NO_3_^−^ species. To gain deeper insight into this difference, the electronic structures and orbital interactions of the two catalysts were examined. The projected density of states (PDOS) of Cu 3d orbitals for pristine α-Cu(Pz)_2_ and β-Cu(Pz)_2_ are shown in Fig. 5a and d. In α-Cu(Pz)_2_, the Cu 3d states are relatively localized, with narrow distributions and weak overlap, indicating limited hybridization with the surrounding ligand orbitals. In contrast, β-Cu(Pz)_2_ exhibits broader and more continuous 3d states, implying enhanced delocalization and stronger Cu-ligand electronic coupling. Upon NO_3_^−^ adsorption, β-Cu(Pz)_2_ displays a more pronounced overlap between the O 2p orbitals of NO_3_^−^ and the Cu 3d orbitals, revealing stronger Cu–O electronic coupling and greater charge transfer capability. This conclusion is further supported by the integrated crystal orbital Hamilton population (ICOHP) analysis, where the Cu–O bonds in α-Cu(Pz)_2_ and β-Cu(Pz)_2_ with adsorbed NO_3_^−^ are −0.185 eV and −0.782 eV (Fig. 5b and e), respectively. The more negative ICOHP value signifies a stronger chemical bonding interaction,^40^ corroborating that the *NO_3_ binds more tightly to β-Cu(Pz)_2_ than to α-Cu(Pz)_2_. The charge density difference maps (Fig. 5c and f) also demonstrate that the NO_3_^−^ species gains approximately 0.555 e and 0.833 e from α-Cu(Pz)_2_ and β-Cu(Pz)_2_, respectively, confirming more significant charge transfer in the latter. Overall, these results reveal that the enhanced NO_3_RR performance of β-Cu(Pz)_2_ originates from its stronger Cu–O orbital hybridization and greater electron delocalization, which can effectively facilitate the NO_3_^−^ activation and reduction.

## Conclusions

In summary, two copper-pyrazolate polymorphs α-Cu(Pz)_2_ and β-Cu(Pz)_2_ were successfully synthesized. Both samples feature 1-periodic chain structures, while their local coordination geometry were dramatically different, with α-Cu(Pz)_2_ adopting a *trans* configuration and β-Cu(Pz)_2_ adopting a *cis* configuration. Electrocatalytic NO_3_RR measurements revealed that the β-Cu(Pz)_2_ demonstrates a markedly superior performance, achieving a FE of 93.33%, compared with 53.10% of α-Cu(Pz)_2_. This enhancement originates from their distinct electronic structures. In β-Cu(Pz)_2_, the Cu 3d orbitals are more delocalized, enabling greater electron donation to the adsorbed *NO_3_ species, thereby promoting the nitrate adsorption, activation, and subsequent reduction. These findings highlight the crucial role of coordination geometry engineering in tuning the electronic properties of active metal centers, providing valuable insights for the rational design of advanced electrocatalysts for sustainable ammonia synthesis and beyond.

## Author contributions

Conceptualization, Zhanning Liu; methodology and investigation, Zhanning Liu, and Shanna An; software, Zhanning Liu, Qingzhong Xue, and Jian Tian; funding acquisition, Zhanning Liu; resources, Qingzhong Xue and Jian Tian. All authors have read and agreed to the published version of the manuscript.

## Conflicts of interest

There are no conflicts to declare.

## Supplementary Material

SC-017-D5SC09385F-s001

## Data Availability

The data underlying this study are available in the published article and its supplementary information (SI). Supplementary information: experimetnal details, DFT calculations, SEM, XPS, electrochemical measurement results, and crystal structure models. See DOI: https://doi.org/10.1039/d5sc09385f.
