# Supplementary material for: Polymorphism-driven coordination geometry engineering for boosting nitrate electroreduction in Cu-pyrazolate chains
Source: Chem Sci. 2026 Mar 9;17(17):8521–7. doi: 10.1039/d5sc09385f (PMC12986422; doi:10.1039/d5sc09385f)
Supplement: SC-017-D5SC09385F-s001 [file SC-017-D5SC09385F-s001.pdf]

Supplementary Information (SI) for Chemical Science.
This journal is © The Royal Society of Chemistry 2026

Supporting Information

Polymorphism-Driven Coordination Geometry Engineering for Boosting Nitrate Electroreduction in Cu–Pyrazolate Chains

Zhanning Liu,[a†*] Shanna An,[a†] Qingzhong Xue,[a*] Jian Tian[a*]

a. School of Materials Science and Engineering, Shandong Key Laboratory of Special Epoxy Resin, Shandong University of Science and Technology, Qingdao, 266590, China.

E-mail: znliu@sdust.edu.cn; xueqz@upc.edu.cn; jiantian@sdust.edu.cn

[†]These authors contributed equally to this work

Experimental details

Synthesis of α-**Cu(Pz)$_2$**

The α-**Cu(Pz)$_2$** was synthesized via a reflux reaction according to the previous reference.[1] 4.06 g copper powder and 5 g pyrazole (HPz) were thoroughly mixed and placed in a 100 mL round-bottom flask equipped with a reflux condenser. The mixture was heated to 110ºC under continuous stirring for 18 h. The resulting green solid was collected by centrifugation, washed several times with deionized water and ethanol, and then dried at 100ºC overnight.

Synthesis of β-**Cu(Pz)$_2$**

The β-**Cu(Pz)$_2$** was synthesized via a coprecipitation reaction at room temperature according to the previous reference.[1] Typically, 2.0 g copper acetate was dissolved in 200 mL acetonitrile to form solution A, while 1.4 g HPz was dissolved in 10 mL acetonitrile to form solution B. Subsequently, solution B was added to solution A under ultrasonic treatment, and a pale pink precipitate formed immediately. The mixture was then allowed to stand for 6 h. The resulting solid was collected by centrifugation, washed several times with deionized water and ethanol, and then dried at 100ºC overnight.

Characterization

Powder X-ray diffraction patterns (PXRD) were collected on a PANalytical diffractometer

using Cu $K\alpha$ radiation. The Rietveld refinements were performed with the GSAS-II package.[2]

The morphology of samples was examined by scanning electron microscope (SEM) on FEI Nova Nano SEM 450 (USA).

X-ray absorption fine structure (XAFS) spectra at the Cu K-edge were collected in transmission mode using the Rapid XAFS (Anhui Absorption Spectroscopy Analysis Instrument Co., *Ltd*, 20 kV and 40 mA). The raw XAFS data were processed using standard procedures with the ATHENA software.[3] The EXAFS data were fitted using the ARTEMIS package.

X-ray photoelectron (XPS) measurements were performed on Thermo ESCALAB 250XI (USA) and calibrated with the C 1s binding energy of 284.8 eV.

In situ FT-IR spectroscopy was performed using a Bruker IFS 125 HR spectrometer. A platinum wire and an Ag/AgCl electrode were used as counter and reference electrodes, respectively. 0.1 M Na_2SO_4 and 0.1M KNO_3 were used as electrolyte with Nafion-117 acting as ion-exchange membrane. The infrared spectra were collected at different potentials ranging from -0.3 V to -1.0 V. Raman spectra were performed on a LabRAM Odyssey system (HORIBA) equipped with a confocal microscope. A 532 nm solid-state diode laser (Coherent Verdi-2) served as the excitation source.

Electrochemical measurements

The finely ground catalysts (5 mg) were dissolved in a water-ethanol solution (1:1 volume ratio, 960 μL). Subsequently, 20 μL of Nafion suspension (5 wt%) was added into the above solution, and the solution was thoroughly mixed by ultrasonic treatment, forming a homogeneous ink. Following this, the catalyst ink was drop-casted onto a glassy carbon electrode with a diameter

of 3 mm and then dried at room temperature. All electrochemical measurements were performed on a CHI 660E electrochemical workstation (Chenhua Shanghai, China). Electrocatalysis reaction took place in a customized H-cell, which was divided into a cathode chamber and an anode chamber by a Nafion 117 membrane. 0.1 M $NaSO_4$ solution containing 0.1 M KNO_3 was used as electrolyte, which was purged with high-purity Ar for 30 min before the measurement. Chronoamperometry tests were performed for 2 h under different potentials. Colorimetric method (indophenol blue method) was used to detect the NH_3 products. Electrochemical impedance spectroscopy (EIS) was recorded at frequencies ranging from 0.1 Hz to 100 kHz. The cyclic voltammetry curves in electrochemical double-layer capacitance (C_{dl}) determination were measured in a non-Faradaic potential window with different scan rates.

Product concentration determination

A 2 mL aliquot of the solution was extracted from the cathode chamber of the H-type electrolytic cell. To this, 2 mL of 1.0 M NaOH solution containing 5 wt.% salicylic acid and 5 wt.% sodium citrate was added, followed by 1 mL of 0.05 M NaClO and 0.2 mL of a 1 wt.% $Na_2[Fe(NO)(CN)_5]\cdot 2H_2O$ aqueous solution. The mixture was left to react for 2 hours at room temperature. Afterward, the UV-vis absorption spectrum was recorded using a UV-2600i spectrophotometer. The absorbance at 655 nm was used to assess the formation of indoxyl blue. A concentration-absorbance calibration curve was established using standard NH_4Cl solutions of varying concentrations.

DFT calculations

The bonding strengths were calculated based on the Vienna *ab* initio simulation package (VASP)[4] in the framework of DFT. The projector augmented wave (PAW) method was used with the Perdew-Burke-Ernzerhof (PBE) generalized gradient approximation (GGA) exchange-correlation energy.[5-7] The crystal structures obtained from Rietveld refinements were used as the raw structural models and were fully optimized until the total energy change was below 1.0×10^{-5} eV and the residual forces on all toms were less than 0.02 eV/Å. A plane-wave cutoff energy of 500 eV and a 3 × 1 × 1 Monkhorst–Pack *k*-point mesh were employed for Brillouin-zone integration. The crystal orbital Hamilton population -COHP and their energy integrals (-ICOHP) were calculated using the local-orbital basis suite toward electronic structure (LOBSTER).[8-9] The free energy change (ΔG) for adsorptions were determined as follows:

$$\Delta G = E_{total} - E_{slab} - E_{mol} + \Delta E_{ZPE} - T\Delta S$$

Where E_{total} is the total energy for the adsorption state, E_{slab} is the energy of the pure surface, E_{mol} is the energy of the adsorption molecule, ΔE_{ZPE} is the zero-point energy change, and ΔS is the entropy change.

As for the adsorption of NO_3^-, to avoid calculating the energy of charged NO_3^- directly, gaseous HNO_3 is chosen as a reference instead. The adsorption energy of NO_3^- (G_{*NO_3}) is described as

$$\Delta G_{*NO_3} = G_{*NO_3} - G_{*} - G_{HNO_3(g)} + \frac{1}{2}G_{H_2} + \Delta G_{correct}$$

where G_{*NO_3}, G_{*}, $G_{HNO_3(g)}$, and $G_{H_2}$are the Gibbs free energy of NO_3^- adsorbed on the catalyst substrates, HNO_3, and H_2 molecules in the gas phase, respectively. $\Delta G_{correct}$ denotes the correction of adsorption energy and is set to 0.392 eV.[10]

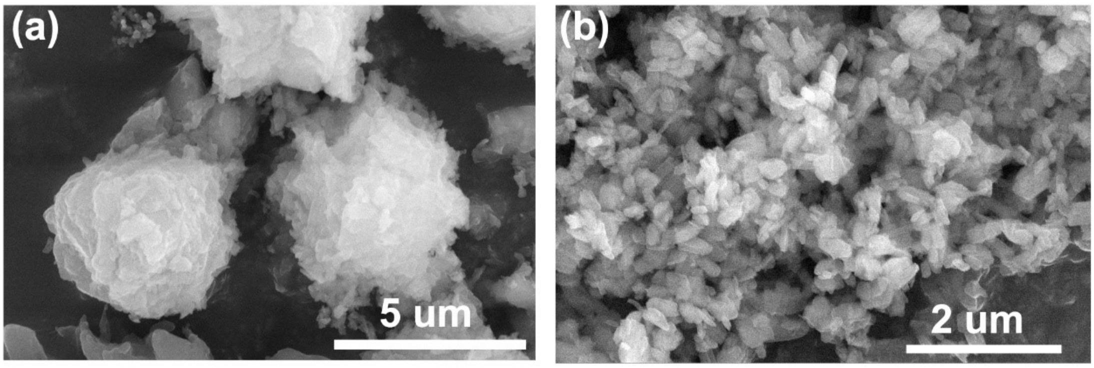

Figure S1 SEM images of (a) ***α*-Cu(Pz)$_2$** and (b) ***β*-Cu(Pz)$_2$**.

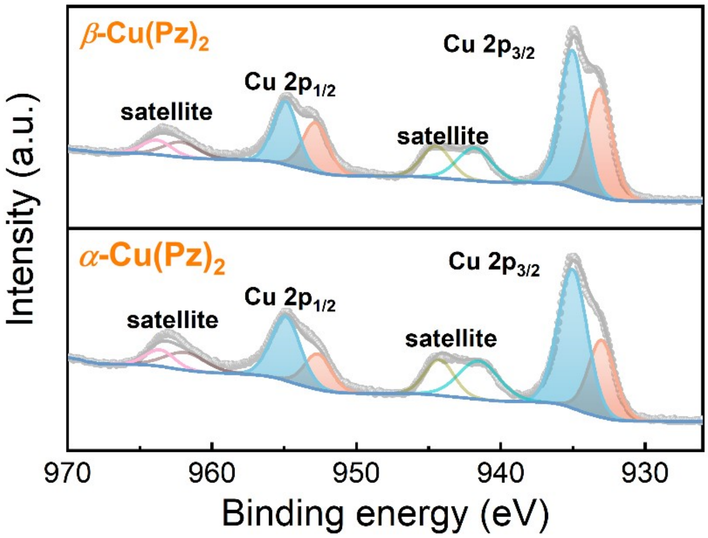

Figure S2 High-resolution XPS spectra of Cu 2p of ***α*-Cu(Pz)$_2$** (bottom) and ***β*-Cu(Pz)$_2$** (top), with corresponding peak assignments.

Table S1 EXAFS fitting results of ***α*-Cu(Pz)$_2$**.

Path	R(Å)	Coordination number	σ^2	ΔE_0	S_0^2	R
Cu-N	1.94	4	0.0015	-19.687	0.95	0.011
Cu-C	2.85	4	0.0024			

Table S2 EXAFS fitting results of ***β*-Cu(Pz)₂**.

Path	R(Å)	Coordination number	σ^2	ΔE_0	S_0^2	R
Cu-N	1.95	4	0.007	-21.74	0.95	0.034
Cu-C	2.87	4	0.005			

Table S3 DFT calculated ground-state energy (E_0) of the two polymorphs.

Phase	E_0 (eV)
α-Cu(Pz)$_2$	-447.07121
β-Cu(Pz)$_2$	-447.19896

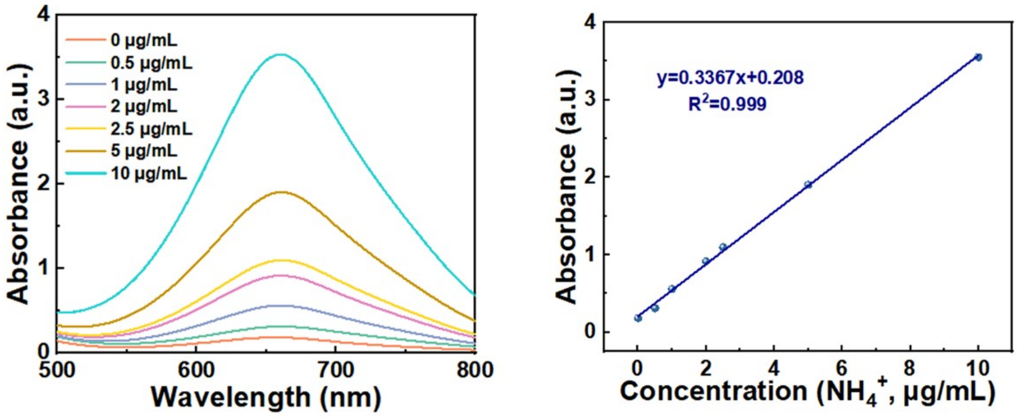

Figure S3 Standard curves for the quantification of NH_3 by the colorimetric method. UV-vis absorption spectra (left) and standard curves of NH_3 at different concentrations (right).

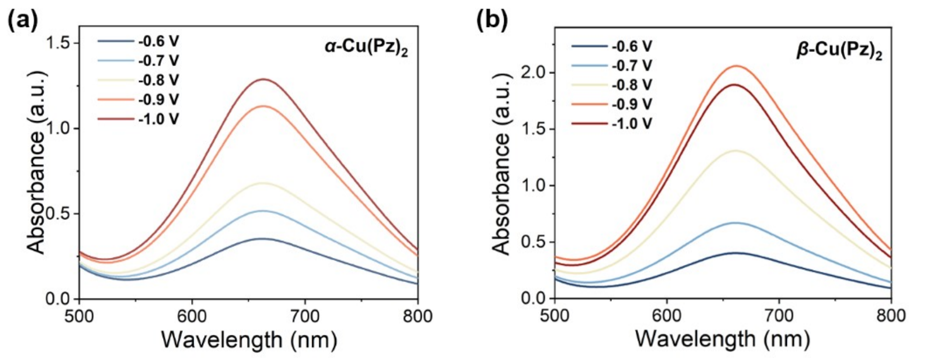

Figure S4 UV-vis absorption spectra of the electrolyte after electrocatalysis at different potentials

for *α*-**Cu(Pz)$_2$** (left) and *β*-**Cu(Pz)$_2$** (right).

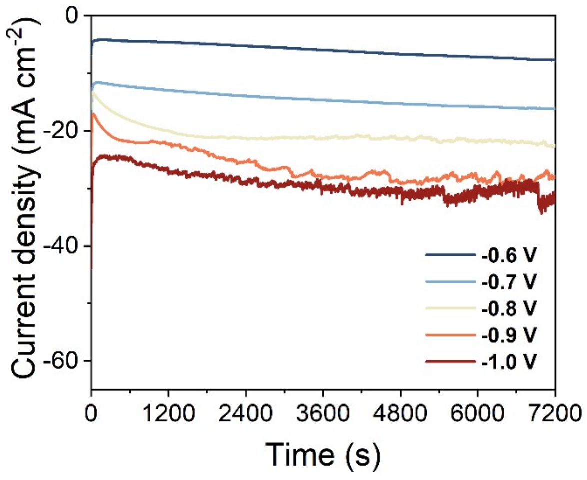

Figure S5 The I-t curves of *β*-**Cu(Pz)$_2$** over 2h.

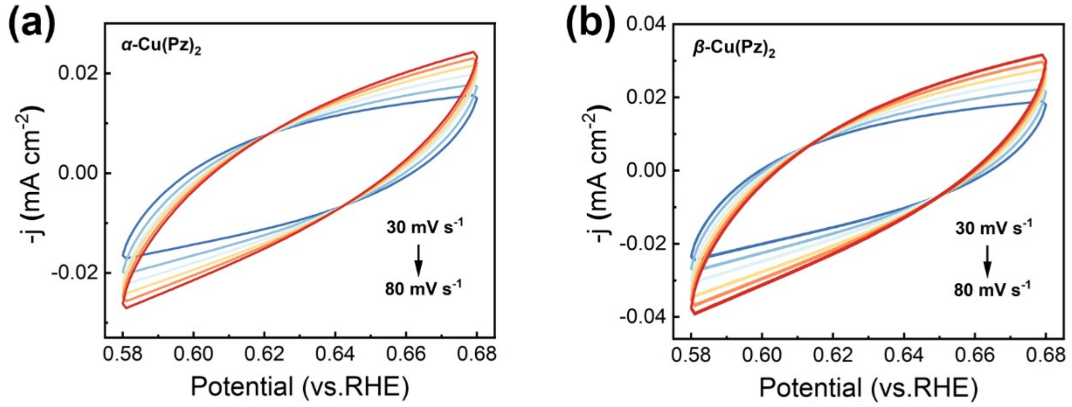

Figure S6 CV curves of *α*-**Cu(Pz)$_2$** (a) and *β*-**Cu(Pz)$_2$** (b) at different scan rates from 30 to 80 mV s^{-1} in the non-Faradic region

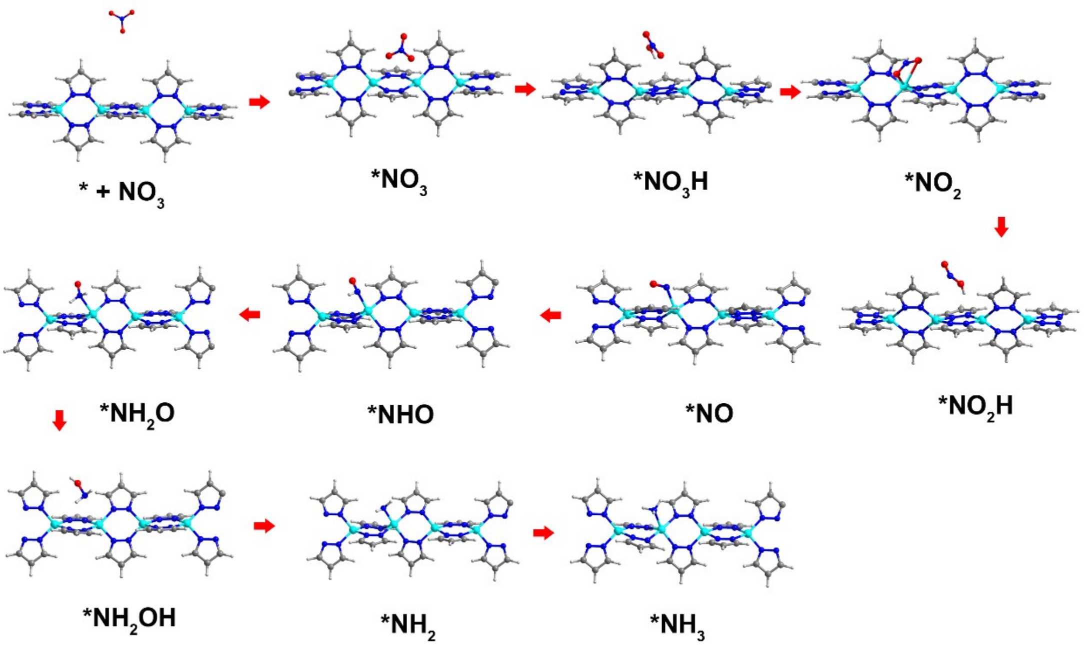

Figure S7 Optimized structural models for NO_3RR pathways on ***α*-Cu(Pz)$_2$.**

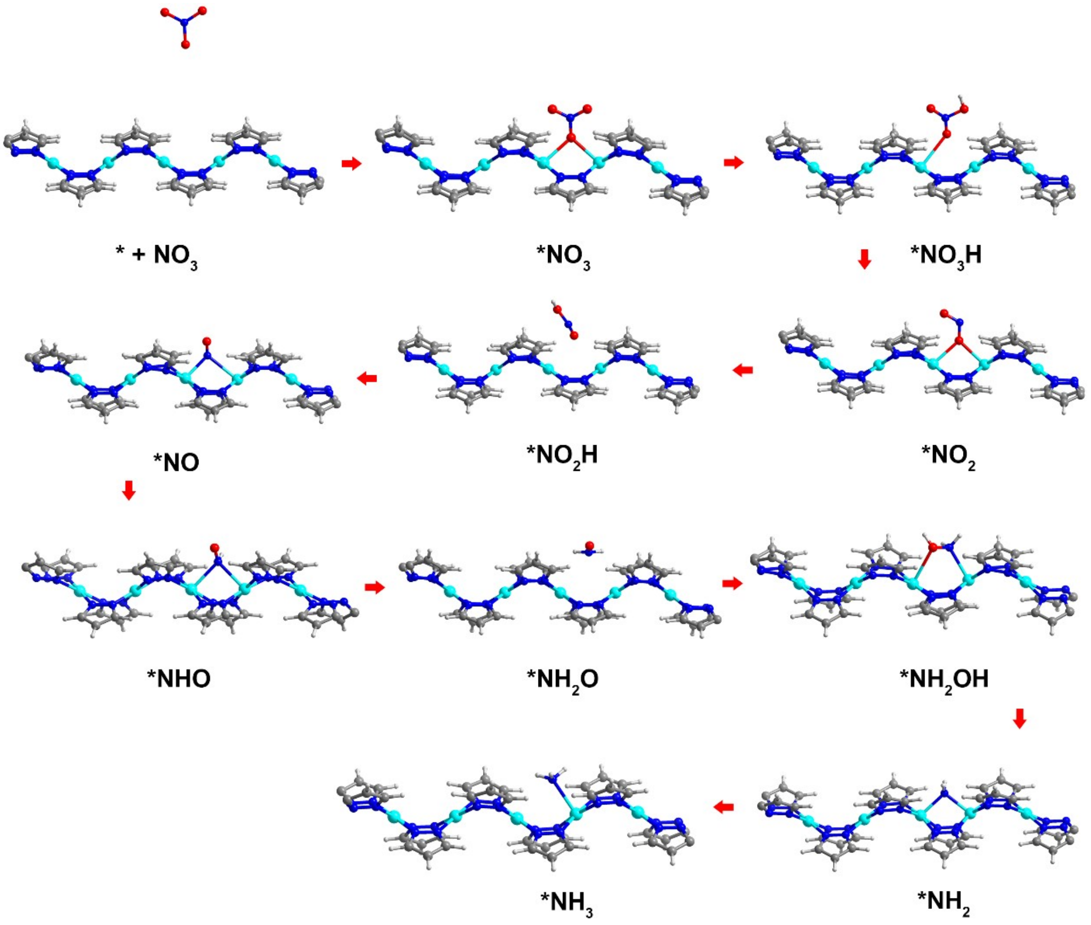

Figure S8 Optimized structural models for NO_3RR pathways on ***β*-Cu(Pz)$_2$**.

Table S4 Some representative reported copper-based electrocatalysts toward NO_3RR.

Catalyst	NH_3 yield rate ($mg\ h^{-1}\ mg_{cat}^{-1}$)	FE (%)	Electrolyte	Electrode	Ref
Rh@Cu	43.18	93	0.1 M KNO_3 0.1 M Na_2SO_4	Carbon cloth	11
Au_1Cu (111)-SAC	0.694	98.7	7.14 mM KNO_3 0.1 M KOH	Carbon paper	12
Cu-N_1O_2 SACs	2.496	96.5	0.01 M KNO_3 and 0.1 M KOH	Carbon paper	13
Pd-CuO	71.4	90	0.1 M KNO_3 1 M KOH	Carbon paper	14
CuO	8.16	95.72	0.1 M KNO_3 0.1 M Na_2SO_4	Glassy carbon	15
CuP2O7	7.33	94.88	0.1 M KNO_3 0.1 M Na_2SO_4	Glassy carbon	15
Cu3(PO4)2	6.53	92.04	0.1 M KNO_3 0.1 M Na_2SO_4	Glassy carbon	15
Cu-BTA	14.32	85.1	0.5 M K_2SO_4 50 mM KNO_3	Carbon cloth	16
Cu-TABQ	18.69	97.7	0.5 M K_2SO_4 50 mM KNO_3	Carbon cloth	16
Cu@Cu-BTC	8.4388	95	0.1 M KNO_3 1 M KOH	Carbon paper	17
UiO-CuZn	-	91.4	200 ppm $NaNO_3$ 0.5 M Na_2SO_4	Carbon paper	18
Cu-CA	3.18	90.3	0.1 M KNO_3 0.1 M Na_2SO_4	Glassy carbon	19
β-Cu(Pz)$_2$	5.5	93.33	0.1 M KNO_3 0.1 M Na_2SO_4	Glassy carbon	This work

Reference

1. A. Cingolani, S. Galli, N. Masciocchi, L. Pandolfo, C. Pettinari, A. Sironi, Sorption− desorption behavior of bispyrazolato− copper (II) 1D coordination polymers. *J. Am. Chem. Soc.* 2005**, 127**, 6144-6145.

2. B. H. Toby, R. B. Von Dreele, GSAS-II: the genesis of a modern open-source all

purpose crystallography software package. *App. Cryst.* 2013**, 46**, 544-549.

3. B. Ravel, M. Newville, ATHENA, ARTEMIS, HEPHAESTUS: data analysis for X-ray absorption spectroscopy using IFEFFIT. *Synchrotron Radiation* 2005**, 12**, 537-541.

4. J. Hafner, Ab-initio simulations of materials using VASP: Density-functional theory and beyond. *J. Comput. Chem.* 2008**, 29**, 2044-2078.

5. P. E. Blöchl, Projector augmented-wave method. *Phy. Rev. B* 1994**, 50**, 17953.

6. M. Ernzerhof, G. E. Scuseria, Assessment of the Perdew–Burke–Ernzerhof exchange-correlation functional. *J. Chem. Phys.* 1999**, 110**, 5029-5036.

7. J. P. Perdew, K. Burke, M. Ernzerhof, Generalized gradient approximation made simple. *Phys. Rev. Lett.* 1996**, 77**, 3865.

8. R. Nelson, C. Ertural, J. George, V. L. Deringer, G. Hautier, R. Dronskowski, LOBSTER: Local orbital projections, atomic charges, and chemical-bonding analysis from projector-augmented-wave-based density-functional theory. *J. Comput. Chem.* 2020**, 41**, 1931-1940.

9. R. Dronskowski, P. E. Bloechl, Crystal orbital Hamilton populations (COHP): energy-resolved visualization of chemical bonding in solids based on density-functional calculations. *J. Phys. Chem.* 1993**, 97**, 8617-8624.

10. H. Niu, Z. Zhang, X. Wang, X. Wan, C. Shao, Y. Guo, Theoretical insights into the mechanism of selective nitrate-to-ammonia electroreduction on single-atom catalysts. *Adv. Funct. Mater.* 2021**, 31**, 2008533.

11. H. Liu, X. Lang, C. Zhu, J. Timoshenko, M. Rüscher, L. Bai, N. Guijarro, H. Yin,

Y. Peng and J. Li, Efficient electrochemical nitrate reduction to ammonia with copper-supported rhodium cluster and single-atom catalysts, *Angew. Chem. Int. Ed.*, 2022, **61**, e202202556.

12. Z.-Y. Wu, M. Karamad, X. Yong, Q. Huang, D. A. Cullen, P. Zhu, C. Xia, Q. Xiao, M. Shakouri and F.-Y. Chen, Electrochemical ammonia synthesis via nitrate reduction on Fe single atom catalyst, *Nat. Commun.*, 2021, **12**, 2870.

13. Z. Gu, Y. Zhang, Y. Fu, D. Hu, F. Peng, Y. Tang and H. Yang, Coordination desymmetrization of copper single-atom catalyst for efficient nitrate reduction, *Angew. Chem. Int. Ed.*, 2024, **136**, e202409125.

14. Y. Liu, Z. Zhuang, Y. Liu, N. Liu, Y. Li, Y. Cheng, J. Yu, R. Yu, D. Wang and H. Li, Shear-strained Pd single-atom electrocatalysts for nitrate reduction to ammonia, *Angew. Chem. Int. Ed.*, 2024, **63**, e202411396.

15. S. An, J. Ren, Y. Xue, J. Tian, In situ construction of Cu^{1+}/Cu^{0} and Cu^{2+}/Cu^{0} pairs of Cu-based catalysts for electrocatalytic nitrate reduction, *Adv. Sci.*, 2025, e17773.

16. R. Zhang, H. Hong, X. Liu, S. Zhang, C. Li, H. Cui, Y. Wang, J. Liu, Y. Hou, P. Li, Z. Huang, Y. Guo and C. Zhi, Molecular engineering of a metal-organic polymer for enhanced electrochemical nitrate-to-ammonia conversion and zinc nitrate batteries, *Angew. Chem. Int. Ed.*, 2023, **62**, e202309930.

17. J. Yu, Y. Qin, X. Wang, H. Zheng, K. Gao, H. Yang, L. Xie, Q. Hu and C. He, Boosting electrochemical nitrate-ammonia conversion via organic ligands-tuned

proton transfer, *Nano Energy*, 2022, **103**, 107705.

18. Z. Wang, S. Liu, M. Wang, L. Zhang, Y. Jiang, T. Qian, J. Xiong, C. Yang and C. Yan, In situ construction of metal–organic frameworks as smart channels for the effective electrocatalytic reduction of nitrate at ultralow concentrations to ammonia, *ACS Catalysis*, 2023, **13**, 9125-9135.

19. C. Xing, J. Ren, L. Fan, J. Zhang, M. Ma, S. Wu, Z. Liu, J. Tian, π-d conjugated copper chloranilate with distorted Cu-O_4 site for efficient electrocatalytic ammonia production, *Adv. Funct. Mater.*, 2024, **34**, 2409064.
